# Retraction: The role of CEUS in characterization of superficial lymph nodes: a single center prospective study

**DOI:** 10.18632/oncotarget.18637

**Published:** 2017-06-27

**Authors:** Giorgio de Stefano, Umberto Scognamiglio, Filomena Di Martino, Roberto Parrella, Francesco Scarano, Giuseppe Signoriello, Nunzia Farella

**Retraction:** Data in this paper was plagiarized from “Clinical application of contrast-enhanced ultrasonography in diagnosis of superficial lymphadenopathy,” in the Journal of Ultrasound in Medicine. 2010; 29:735-40.

**Footnotes:** The online version of the original article can be found under doi.

Original article: Oncotarget. 2016; 7:52416-52422. doi: 10.18632/oncotarget.9385

